# Blood Lipids and Cognitive Performance of Aging Polish Adults: A Case-Control Study Based on the PolSenior Project

**DOI:** 10.3389/fnagi.2020.590546

**Published:** 2020-11-17

**Authors:** Oliwia McFarlane, Mariusz Kozakiewicz, Kornelia Kędziora-Kornatowska, Dominika Gębka, Aleksandra Szybalska, Małgorzata Szwed, Alicja Klich-Rączka

**Affiliations:** ^1^Department of Social and Medical Sciences, L. Rydygier Collegium Medicum in Bydgoszcz, Nicolaus Copernicus University in Toruń, Toruń, Poland; ^2^Department of Geriatrics, L. Rydygier Collegium Medicum in Bydgoszcz, Nicolaus Copernicus University in Toruń, Toruń, Poland; ^3^Department of Geriatrics, Division of Biochemistry and Biogerontology, L. Rydygier Collegium Medicum in Bydgoszcz, Nicolaus Copernicus University in Toruń, Toruń, Poland; ^4^International Institute of Molecular and Cell Biology, Aging and Longevity Strategic Project, Warsaw, Poland; ^5^Department of Human Epigenetics, Mossakowski Medical Research Centre, Polish Academy of Sciences, Warsaw, Poland; ^6^Department of Internal Medicine and Gerontology, Faculty of Medicine, Jagiellonian University Medical College, Kraków, Poland

**Keywords:** neurodegeneration, mild cognitive impairment, dementia, cognition, cholesterol, biomarker, 24(S)-hydroxycholesterol, plasma

## Abstract

**Background**: The demand for effective strategies for maintaining cognitive capableness and establishing early dementia diagnosis has been tremendous, especially in the context of population aging. However, studies on the elderly population and neurocognitive impairment had provided ambiguous results throughout, while potential blood biomarkers of cognitive decline are yet to be clearly understood.

**Objectives**: The present study is aimed at assessing the relationship between blood lipids—especially in the context of their usefulness as biomarkers of an early cognitive decline—and cognitive functioning of aging adults.

**Materials and Methods**: The study sample consisted of 230 participants—(109 women, 121 men) aged 65+ years. Plasma 24(S)-hydroxycholesterol [24(S)-OHC], serum total cholesterol (TC), high-density lipoprotein cholesterol (HDL), and low-density lipoprotein cholesterol (LDL) were assessed. The analyses were conducted in three groups of cognitive performance: cognitively normal, mild cognitive impairment (MCI), and mild dementia, of which the subjects were divided with the Mini-Mental State Examination (MMSE).

**Results**: No significant differences in 24(S)-OHC plasma concentrations for different levels of cognitive performance were found. Significant differences were found in serum TC (*p* = 0.026) and LDL (*p* = 0.007) concentrations for different levels of cognitive performance. Concentrations of both parameters were highest in the MCI group and lowest in mild dementia and cognitive norm, respectively. No significant differences between serum HDL concentrations and cognitive performance were found.

**Conclusions**: To fully assess the potential of research on blood lipids in regards to a cognitive decline, cross-sectional or epidemiological studies aimed at further exploring blood lipid roles in both the early and advanced MCI and dementia, are needed.

## Highlights

- No differencesNo differences in 24(S)-OHC plasma concentrations for different levels of cognitive performance were observed
- No differencesConcentrations of both total cholesterol and LDL fraction were highest in the MCI group
- No differences24(S)-OHC cannot be qualified as a blood biomarker of an early neurodegeneration

## Introduction

Cholesterol is a pivotal constituent of neurons, and the brain is its most substantial source, sustaining about 25% of all cholesterol in the body (Dietschy and Turley, 2001). About 70% is found in myelin (Petrov et al., 2016), and around 30% in membranes of neuronal and glial cells, where it is metabolically active and undergoes recycling for neuron repair and remodeling (Dietschy and Turley, 2004). It constitutes a crucial component of cell membranes (Herz and Bock, 2002), and myelin sheath which provides insulation for the transmission of nerve impulses, where its loss inevitably causes neurological difficulties. It influences the functioning of brain synapses and is pivotal in both production, and secretion of neurotransmitters. A connection allying cholesterol metabolism deficiencies and neurodegenerative processes has been acknowledged (Petrov et al., 2016).

The initial studies on cholesterol and cognition by Sparks et al. (1990, 1994) sparked numerous research on cholesterol and cognitive impairment, some suggesting that hypercholesterolemia may be an important risk factor of neurodegenerative diseases (Liu et al., 2016). Research showed that demented or cognitively impaired aging adults show an increase in plasma total cholesterol (TC) or low-density lipoprotein cholesterol (LDL), as compared to non-demented individuals of the same gender and age (Evans et al., 2000; Lesser et al., 2001; Yaffe et al., 2002). Several, although—in most cases—retrospective epidemiological studies, mentioned a smaller rate of Alzheimer’s disease (AD) amongst patients taking statins (Jick et al., 2000; Wolozin et al., 2000, 2007; Cramer et al., 2008; Haag et al., 2009). Stronger evidence, causally connecting cholesterol with AD was revealed by experimental studies indicating that controlling the amount of this lipid, altered the concentrations of amyloid precursor proteins (APP) and beta-amyloid (Friedhoff et al., 2001). Nevertheless, several uncertainties regarding the supposition causally connecting cholesterol with AD have emerged. Some studies failed to observe the discrepancies or indicated that cholesterol concentrations in AD patients were lower than non-demented individuals (Romas et al., 1999; Slooter et al., 1999; Kalmijn et al., 2000; Knittweis and McMullen, 2000; Tan et al., 2003), reporting negative correlations of serum lipid values with AD (or with all dementias; Solfrizzi et al., 2002; Mielke et al., 2005). Also, prospective studies on statins and AD have not entirely supported those associations (Rea et al., 2005; Zandi et al., 2005; McGuinness et al., 2013). Clinical interventions manipulating statins to lower cholesterol levels to prevent and cure neurodegeneration proved unsuccessful (McGuinness et al., 2014). Even experimental data is open to different interpretations, as modifying cholesterol levels impacts various proteins, not confined to APP and beta-amyloid (Wood et al., 2014). Additionally, peripheral cholesterol cannot cross the blood-brain barrier (BBB; Björkhem and Meaney, 2004). The aforementioned presumptions do not validate the causing role of elevated cholesterol concentrations in AD or mild cognitive impairment (MCI; Liu et al., 2016). At present, molecular mechanisms constituting neurodegeneration remain unclear and we have no valid blood indicators for initial diagnosis. The inconsistency of the findings of numerous up-to-date scientific investigations suggests that added efforts aimed at the observation and analysis of lipid measurements are needed, especially as blood-based biomarkers are easily acquired, relatively inexpensive, and more feasible for extensive application than cerebrospinal fluid (CSF) markers.

Over the years, the variable that began being associated with neurodegenerative processes is the modified metabolism of brain cholesterol, with research suggesting that oxidized derivatives of cholesterol—oxysterols (OHC)—might be amongst the most important determinants of AD. They are biologically active cholesterol metabolites circulating in plasma that may be formed enzymatically or by autoxidative mechanisms (Dias et al., 2018). Oxysterols, such as 27-OHC, 24S-OHC, 7α-OHC i 7β-OHC not only penetrate the BBB but exhibit cytotoxic and proapoptotic potential. Among those, 24S-OHC is the most prevalent in the brain. It modulates cholesterol homeostasis and aids neuronal activity through the activation of liver X receptors (Okabe et al., 2013). The elevation of 24S-OHC in plasma in the course of neurodegenerative disorders is believed to show neuronal atrophy and growing secretion to the circulation; therefore, it is possibly mostly related to neurocognitive disorder pathogenesis (Testa et al., 2016).

### 24(S)-hydroxycholesterol [24(S)-OHC]

The conversion of cholesterol to 24S-OHC is catalyzed by cholesterol 24-hydroxylase (CYP46A1), abundantly expressed in the brain (Boussicault et al., 2016). Plasma concentration of 24(S)-hydroxycholesterol [24(S)-OHC] depends on various determinants, including cholesterol turnover factors, liver oxysterol elimination, plasma lipoprotein metabolism, genetics, and behavioral patterns (Leoni and Caccia, 2013). It has been hypothesized that plasma levels of 24(S)-OHC can serve as the first biochemical indicators of modified homeostasis of cholesterol in the central nervous system (CNS; Lütjohann et al., 2000). Further research delivered contradictory outcomes, reporting normal (Schönknecht et al., 2002) or lowered (Bretillon et al., 2000; Kölsch et al., 2004; Solomon et al., 2009) levels of 24(S)-OHC in demented vs. cognitively normal individuals. Some studies indicate that plasma concentrations of 24(S)-OHC may be higher in initial AD and Vascular dementia (VaD), possibly due to cholesterol turnover elevation associated with neuronal degradation or a defect in the BBB, present in neurodegenerative disorders, including AD (Zuliani et al., 2011). BBB defects, the occurrence of inflammation, or elevated cholesterol turnover can all counterbalance this notion, causing an increase in, or—occasionally—modification of 24(S)-OHC plasma concentrations. Similarly, reduction of those levels in severe dementia can be associated with the deficit of metabolically active neurons and the atrophy level of cell membranes (Schönknecht et al., 2002). Scarce work has focused on the evaluation of the associations between oxysterols and MCI (Liu et al., 2016). Initial reports suggest that increased concentrations of 24(S)-OHC (Leoni et al., 2006) or 27(S)-hydroxycholesterol (27-OHC; Liu et al., 2016) in CSF can pose a neurodegeneration indicator in individuals with MCI.

## Materials and Methods

The study was conducted with retrospective use of selected data and biological material from a nationwide cross-sectional PolSenior project carried out between 2007 and 2012. The project originally involved 5,695 respondents, of which 4,979 were aged 65 years and over, and 716 aged 55–59 years. Participants had been subject to randomized selection from 16 administrative zones of Poland by a multi-stage, proportional, age-group stratified process, as outlined previously (Bledowski et al., 2011). The study fully complied with all applicable institutional and governmental regulations concerning the ethical use of human volunteers, and with the terms of the Helsinki Declaration. The institutional review board approved the study protocol (the Bioethics Committee of the Medical University of Silesia in Katowice, Poland; no KNW-6501-38/I/08) and all the recruited subjects gave their written informed consent.

### Participants

The study sample consisted of 230 subjects, including 109 women and 121 men, aged 65+ years selected amongst the PolSenior project respondents from the kujawsko-pomorskie voivodeship. The sampling followed strict exclusion criteria: (1) statin ingestion; (2) symptoms of depression—Geriatric Depression Scale 15-item version (GDS-15) >5 points; (3) moderate or severe dementia; (4) brain stroke; and (5) other pathological states that could severely influence cognitive functioning. To ensure comparability, analyses were conducted in three age- and gender-matched groups of cognitive performance: cognitively normal, MCI, and mild dementia. Cognitive function was screened using the Mini–Mental State Examination (MMSE; Folstein et al., 1975). The total score of MMSE is 30 points, of which 30–28 points were considered as the cognitive norm, 27–24 points as MCI, and 23–20 points as mild dementia. The test had been administered by trained nurses. The overall cognitive score was based on the MMSE result alone.

### Biochemical Parameters

Blood samples were obtained from each participant during a home visit by vacuum venipuncture to ensure transport safety. Samples were delivered by nurses within a maximum of 2 h to project local laboratories, where after separating serum and plasma they were stored at −20°C until analysis. Serum concentrations of TC (measurement range: 3–800 mg/dl; error: <1.7%), LDL (measurement range: 3–550 mg/dl; error: <1.2%) and high-density lipoprotein (HDL; measurement range: 3–120 mg/dl; error: <1.3%) were assayed in the Central Laboratory in Warsaw with the use of an enzymatic colorimetric method (Modular PPE, Roche Diagnostics, Mannheim, Germany). Plasma concentrations of 24(S)-OHC (test sensitivity: 0.78 ng/ml) were assayed in the Department and Clinic of Geriatrics Collegium Medicum in Bydgoszcz with the use of the ELISA immunoenzyme assay.

### Functional Assessment

We conducted a retrospective analysis of respondent data, including chosen clinical parameters (age, gender, socioeconomic data, health, and lifestyle), and results of GDS-15 (Sheikh and Yesavage, 1986), Katz Index of Activities of Daily Living (ADL; Katz et al., 1970), and Lawton Instrumental Activities of Daily Living Scale (IADL; Lawton and Brody, 1969), which is reported in the sample description below.

### Statistical Analyses

The main analyzed variable regarding cognitive functioning level was the MMSE test result. It was explored regarding both plasma 24(S)-OHC and serum lipid levels, with the use of appropriate statistical methods, as detailed below. The functional assessment (e.g., chosen clinical parameters, ADL, and IADL test results) was used to describe the study sample. Statistica 10.0 (StatSoft, Inc., 2016), R statistical packet (R Core Team, 2016) and RStudio environment (RStudio Team, 2016) were used for all analyses. Normally distributed data (serum lipid levels) are presented as mean ± standard deviation and were analyzed using the independent-samples *t*-test or ANOVA with the RiR Turkey test, as appropriate. Not-normally distributed data (plasma lipid levels) are presented as median with one and three quartiles and were analyzed using the Mann–Whitney *U* test or the Kruskal–Wallis test, as appropriate. Correlations were assessed using the Spearman Rank correlation test for nonparametric distribution. *P*-value <0.05 was considered to indicate statistical significance.

## Results

### Demographic Characteristics and Functional Assessment

Cognitively normal, referred to as control group, consisted of 71 subjects (33 women and 38 men, with the average age of 77.8); MCI group consisted of 85 participants (43 women and 42 men, with the average age of 78.8); mild dementia group consisted of 74 subjects (33 women and 41 men, with the average age of 80.7). The study sample was age-and gender-matched. The sample was homogenous regarding the assessed socioeconomic, lifestyle, and health factors that could affect cognitive performance. The vast majority (78%) achieved the primary or vocational education level. Ninety-seven percentage were employed at some part of their lives, where 71% were physical laborers or farmers. Seventy-six percentage received their financial situation as good. The sample was very homogenous in terms of having children—95% had them. Seventy-six percentage reported undertaking activities requiring physical activity in the last 12 months, however, the group was not physically active in general. Current participation in exercise or rehabilitation was declared by only 13% of the respondents, and exercising or undertaking sports activities in the past–14%. The ability to climb the 1st floor was declared by 89%, while 13% thought they could swim 10 m. The majority declared good health; 18% had the legal title of being disabled; 87%—normal thyroid function, and the remaining—subclinical thyroid disease; 73%—no vision problems. Ninety-five percentage did not report a history of depression treatment. Eighty-four percentage declared their alcohol intake to be not more than a few servings a year. Eighty-four percentage of respondents were completely independent in basic ADLs. The most differentiating variables were the declared ability to ride a bike (59% non-riders and 41% riders), smoking (43% of former/current smokers vs. 57% of non-smokers), and independence in instrumental ADL, declared by only 53% of the respondents (Tables s 1, 2).

**Table 1:** Activities of daily living (ADL) results of the study sample.

ADL questionnaire points	Number	Percent
0–2	3	1.3
3–4	3	1.3
5–6	224	97.4

*ADL interpretation: 5–6 points: no disorders. 3–4 points: moderate impairment. 0–2 points: severe impairment*.

**Table 2:** Instrumental activities of daily living scale (IADL) results of the study sample.

IADL questionnaire points	Number	Percent
8–18	44	19.3
19–23	63	27.6
24	121	53.1

*IADL interpretation: 8–18 points: severe dependence. 19–23 points: moderate dependence. 24 points: full independence*.

### Plasma Concentrations of 24(S)-OHC

No statistically significant differences were found in 24(S)-OHC concentrations for different groups of cognitive performance. No statistically significant differences were found in 24(S)-OHC concentrations based on gender (Tables s 3, 4).

**Table 3:** Plasma 24(S)-OHC levels [median (1 quartile–3 quartile)] and cognitive performance measured with the Mini-Mental State Examination (MMSE).

	Cognitive functioning level			
	Norm	MCI	Mild dementia	K–W *p* level
24(S)-OHC	199.7	209.8	200.2	0.225
	[184–222.1]	[193.3–224.3]	[178.6–216.3]	

**Table 4:** Plasma 24(S)-OHC levels [median (1 quartile–3 quartile)] and gender.

	Gender		
	Women	Men	U M–W *p* level
24(S)-OHC	206.0 [182.2– 225.1]	201.9 [184.0– 218.4]	0.503

### Serum Concentrations of TC, LDL, and HDL

Statistically significant differences were found in serum TC (*p* = 0.026) and LDL (*p* = 0.007) concentrations for different levels of cognitive performance. No statistically significant differences between serum HDL concentrations and cognitive performance were found (Table 5). We found no statistically significant correlations between 24(S)-OHC and TC, LDL, HDL.

**Table 5:** Serum total cholesterol (TC), low-density lipoprotein cholesterol (LDL), and high-density lipoprotein cholesterol (HDL) concentrations (mean ± SD) with cognitive performance measured with the MMSE.

	Cognitive functioning level			
	Norm	MCI	Mild dementia	ANOVA *p*-level
TC	208.6 ± 42.3	222.4 ± 47.1	205.0 ± 38.0	0.026
LDL	126.5 ± 37.1	142.1 ± 38.4	125.9 ± 32.6	0.007
HDL	48.6 ± 12.3	48.6 ± 12.5	52.5 ± 15.1	0.109

**Figure 1:**
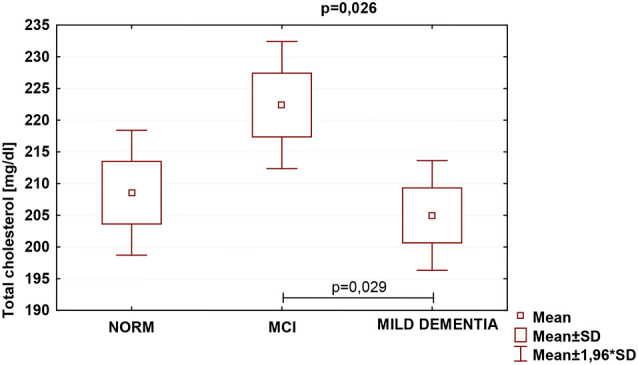
Serum total cholesterol (TC) concentrations and cognitive performance.

**Figure 2:**
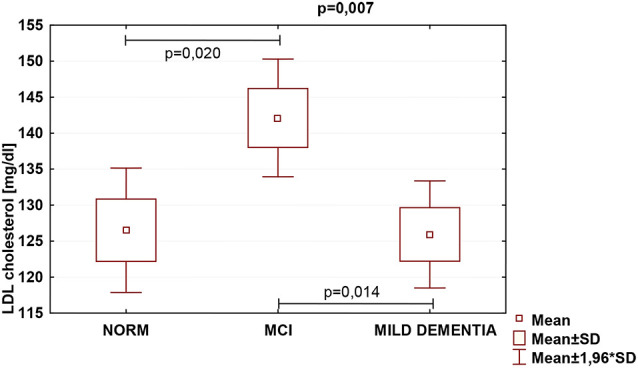
Serum low-density lipoprotein cholesterol (LDL) concentrations and cognitive performance.

**Figure 3:**
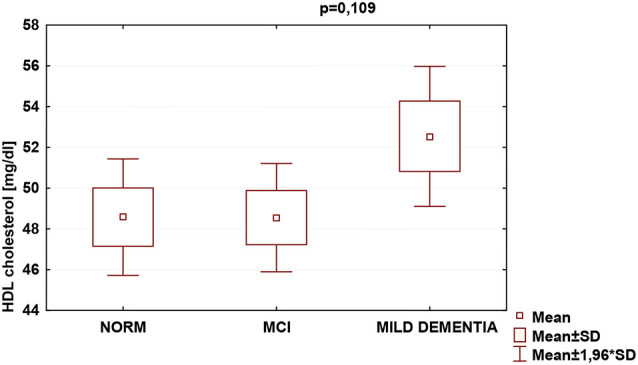
Serum high-density lipoprotein cholesterol (HDL) concentrations and cognitive performance.

TC was the highest in the MCI group (222.4 ± 47.1), and lowest in mild dementia (205.0 ± 38.0). In the cognitive norm, it was 208,6 ± 42,3. LDL concentration was the highest in the MCI group (142.1 ± 38.4), and lowest in cognitive norm (125.5 ± 37.1). In the mild dementia group, the LDL level was 125.9 ± 32.6. Multiple regression revealed statistically significant differences in LDL levels between the cognitive norm and MCI (*p* = 0.020), and MCI and mild dementia (*p* = 0.014). For TC, statistically important differences occurred between MCI and mild dementia only (*p* = 0.029; Table 6, Figures 1–3). We found no statistically significant correlations between 24(S)-OHC and TC, LDL, HDL (Table 6).

**Table 6:** Spearman correlations between 24(S)-OHC and TC, LDL, HDL.

Parameter	24(S)-OHC
TC	−0.01494
LDL	0.01217
HDL	0.02951

## Discussion

Cholesterol homeostasis disorders can impact dementia progression (Liu et al., 1998; Björkhem, 2006). As mentioned before, plasma levels of 24(S)-OHC can reflect brain cholesterol catabolism and in consequence—ongoing neurodegeneration. Therefore, possibly the plasma concentrations of this parameter could be a candidate biochemical marker of altered homeostasis of cholesterol in CNS (Lütjohann et al., 2000). Studies on plasma 24(S)-OHC levels in the context of dementia have brought conflicting results throughout (Bretillon et al., 2000; Lütjohann et al., 2000; Schönknecht et al., 2002; Kölsch et al., 2004; Solomon et al., 2009) with an interesting hypothesis of higher concentrations of brain cholesterol when neurodegeneration markers are elevated, but neuron loss and brain atrophy degree remains small (Zuliani et al., 2011). This is following other clinical observations (Kölsch et al., 2004; Solomon et al., 2009), also indicating that lowered plasma 24(S)-OHC levels are typical for dementia (Liu et al., 1998; Leoni and Caccia, 2013), but they lower gradually with disease progression (Björkhem and Meaney, 2004). According to this hypothesis, we expected 24(S)-OHC levels to be elevated in the mild dementia group in comparison with the cognitive norm; however, this assumption was not confirmed. Perhaps more than one measurement in the course of the disease is required to capture its progression reflected by plasma lipid levels. It was previously demonstrated that plasma levels of both 24-OHC and 7-OHC, but not 27-OHC were higher in demented vs. non-demented individuals, and might be lowered by simvastatin (Vega et al., 2004; Vega and Weiner, 2007).

Few studies assessed correlations between oxysterols and MCI (Tan et al., 2003; Liu et al., 2016), providing conflicting results of either elevated or lowered plasma 24(S)-OHC levels (Lütjohann et al., 2000; Papassotiropoulos et al., 2000). Again, the hypothesis of higher plasma levels of 24(S)-OHC in an early MCI, compared to significant neuron loss stage has been raised (Hughes et al., 2013), for contradictory results might have been caused by research groups with varying MCI duration. Advanced disease was associated with neuronal atrophy and decrease of 24S–OHC, while its onset—with the elevated level of this oxysterol. Possibly the differences resulted from cholesterol release due to the myelin breakdown (Hughes et al., 2013). It seems plausible, since conflicting research, e.g., study by Liu et al. (2016) that found no significant differences in plasma concentrations of 24(S)-OHC between individuals with and without MCI, did not allow to observe the impairment progression. Similarly, our case-control study showed no significant differences in 24(S)-OHC concentrations amongst cognitively normal, MCI and mild dementia patients, thus failing to demonstrate an indicative role of this oxysterol for MCI. There are, therefore, certain premises that lowering plasma levels of 24(S)-OHC can be related to cognitive impairment progression, characterized by loss of metabolically active neurons. According to this hypothesis, elevated cholesterol turnover would counteract this tendency (Schönknecht et al., 2002). However, to test it, prospective studies with multiple measurements of this oxysterol are needed.

Blood lipids can directly affect neurodegeneration (Bretillon et al., 2000; Schönknecht et al., 2002; Kölsch et al., 2004; Li et al., 2005; Fischer et al., 2006; Hall et al., 2006; Reitz et al., 2008; Solomon et al., 2009). Serum TC levels were significantly higher in MCI than in mild dementia, and serum LDL levels were significantly higher in MCI than in cognitively normal and mild dementia groups. HDL levels did not vary significantly between the study populations. Our findings of concentrations of both TC and LDL fraction highest in the MCI group might indicate that this diagnostic entity should be further explored in concerning blood lipid levels. Because those can be altered by eating habits, physical activity, pharmaceuticals, and modification of harmful behaviors, such as tobacco use, the above results might carry major public health implications. Approaches aiming at modification of blood lipid levels may therefore pose feasible large-scale interventions intended for preserving brain function over the years.

So far, findings from investigations on serum lipid levels and cognitive performance have lacked consistency (Kivipelto et al., 2001; Michikawa, 2003; Li et al., 2004; Mielke et al., 2005), similarly to the results of studies on relationships between advanced age lipids with cognitive decline or dementia. We found TC levels significantly different between the study populations; they were lowest in mild dementia, which supports earlier reports on lower TC levels in demented patients (Kuusisto et al., 1997; Panza et al., 2006), as compared to non-demented individuals, even upon completion of a 26-year follow-up (Stewart et al., 2007). The prevailing view, however, has been that elevated cholesterol constitutes a risk factor for dementia. Substantial data have confirmed that both high risk of AD development (Patterson et al., 2008), and considerably increased likelihood of MCI (Piguet et al., 2003), are associated with high TC (Patterson et al., 2008) regardless of possible interfering factors, being indicative of playing a role in developing cognitive impairment (Piguet et al., 2003). In our sample, the highest TC levels were indeed found in the MCI group, which seems to favor this notion. Nonetheless, the process by which high TC could contribute to cognitive decline remains uncertain. Previous data suggested both that modifications of brain cholesterol homeostasis might be associated with the core pathological characteristics of AD, specifically beta-amyloid (Burns and Duff, 2002; Sponne et al., 2004), and that elevated TC in advanced age does not correlate with any form of cognitive decline or dementia (Kivipelto et al., 2001; Beydoun et al., 2011; Anstey et al., 2017). On the contrary, it seems to be linked to a reduced probability of incident AD in older adults (Kuusisto et al., 1997), acting as a preventive measure against cognitive impairment (Piguet et al., 2003). Rather, mid-age elevated levels of TC are connected with an increased odds ratio for MCI and cognitive deterioration in old age (Anstey et al., 2017). The explanation of those discrepancies might partly lie in the moment of cholesterol level assessment regarding the clinical onset of dementia, indicating that TC starts decreasing years before the manifestation of the symptoms, possibly due to ongoing neurodegeneration. This study, providing a one–time measurement of blood lipids in advanced-age individuals, and thus not allowing to observe cholesterol dynamics in the sample over years, warns against drawing bold conclusions regarding the potential of research on TC regarding cognitive decline. However, our findings of lowest TC in mild dementia, medium in the cognitive norm and highest in MCI seem to partly support the above interpretation.

Regarding cholesterol fractions, an Indian study, assessing lipid profiles in similar groups of cognitive functioning, reported AD patients with higher levels of LDL, and lower HDL (Vasantharekha et al., 2017). Additionally, a longitudinal study on 1,159 elderly Chinese revealed correlations of both high TC and LDL concentrations with rapid cognitive deterioration (Ma et al., 2017). It is partially consistent with our findings of the highest levels of both TC and LDL in the MCI group, and lowest LDL in the cognitive norm, which was expected. As to HDL levels, they did not differ significantly between the groups. We find it surprising, a majority of transverse investigations which involved individuals aged 75 plus, did recognize a link between high HDL levels and improved results of cognitive ability examinations (van Vliet et al., 2009). Some recent studies, however, failed to demonstrate both advanced age HDL and triglyceride correlation with a high probability of developing VaD, and HDL with MCI, AD, or other dementia disorders, respectively (Anstey et al., 2017). It ought to be noted that conflicting findings might be derivatives of differences in study and population type selected, age of participants, sampling, diagnostic procedures, methods and measures used, scientific rigor applied, making the results inappropriate for direct comparisons. Consequently, even though research on cholesterol and cognition was initiated decades ago, the associations between blood lipids and cognition are not yet fully understood (Li et al., 2018). We believe that this study with its stringent exclusion criteria, allowed to eliminate the plethora of confounding factors regarding cognitive functioning, thus constituting a valuable reference for further debate.

The study sample was homogenous concerning the majority of the analyzed socioeconomic, lifestyle, and health factors which could impact cognitive functioning (except for smoking and riding a bike). Therefore, it seems justified to test a hypothesis of cognitive functioning level differences resulting from other uncontrolled variables, such as diet or psychological factors. Limitations to this study, mainly resulting from the nature and design of the PolSenior project, have to be taken into consideration; as a cross-sectional, multicenter, community-based project involving nearly 6,000 participants, only routine screening examination of cognition, mood, and functional dependence was performed. Although providing a multi-aspect assessment to a sample this size, representative for the elderly population of a country, definitely constitutes a significant scientific achievement, it also contributes to certain problems, including those of a methodological nature. No results of further detailed assessments are available, including brain imaging data. Findings of this case-control study on aging Polish adults need to be confirmed in further longitudinal studies, and cannot be generalized to other populations. Even though discrepancies amongst study populations have been noted for various lipid parameters, the numerical differences are small. Perhaps population type, diagnostic procedure, or statistical significance could influence data precision. To seize the overall impact of blood lipids on cognitive capabilities more accurately, longitudinal studies spanning over middle-aged individuals and seniors, on several constant lipoproteins and cognitive measures, are necessary. Also, usage of MMSE, not entirely adjusted to the Polish population of the older adults, lacking norms for under-educated individuals, can potentially lead to research bias of underestimating the cognitive performance in a part of the sample. Under-educated individuals might have scored lower as a result of their lack of general knowledge and overall poor level of cognitive performance, which, for them is normal functioning, not indicative of cognitive impairment. This may apply to 26 respondents, constituting 12% of the study sample, who achieved only incomplete primary education, including two people with no education, being probably self-educated in terms of reading, writing, and counting. An interesting for the analyses is the fact that, to our best knowledge, there are no MMSE norms for the Polish population, defined by the sum of points on the scale, constituting cut-off points for subsequent levels of cognitive impairment severity. Determining the ceiling for the under-educated—mostly due to the historical factors—the older population of Poland requires further discussion and research. Application of a full battery of neuropsychological tests would enable a comprehensive cognitive assessment and improve its accuracy, however, the nature and range of the project impeded it. Therefore, although the understanding of blood lipid profiles in the study population is not thorough, they may serve as a base for further exploration, setting out the framework and reference for future research. Despite limitations, it is one of few studies thoroughly investigating blood lipid roles of both circulating and brain lipids concerning the cognitive functioning of the elderly in Poland, also addressing potential blood biomarkers of an early cognitive decline among aging adults.

## Conclusion

Following the criteria for potential blood biomarkers of early neurodegeneration, we can assume that 24(S)-OHC cannot aspire to a biomarker of an early cognitive decline in the elderly. To fully assess the potential of research on cholesterol in the context of a cognitive decline, longitudinal cross-sectional studies aimed at further exploring blood lipid profiles in both the initial and advanced MCI and dementia, are needed. Upcoming studies dedicated to determining the processes that constitute the foundation of the influence of plasma lipids on cognition are hoped to deliver a pivotal view on the triggers and interrelationships of MCI and dementia, as well as prompt original approaches for managing these conditions.

## Data Availability Statement

The raw data supporting the conclusions of this article will be made available by the authors, without undue reservation.

## Ethics Statement

The studies involving human participants were reviewed and approved by the Bioethics Committee of the Medical University of Silesia in Katowice, Poland. The patients/participants provided their written informed consent to participate in this study.

## Author Contributions

OM contributed to the design, analysis of data, and wrote the main manuscript text. MK contributed to the design of work and assayed biochemical parameters. KK-K made substantial contributions to drafting and revising the work. DG contributed to acquisition and interpretation of data. AS made substantial contributions to drafting the manuscript. MS made substantial contributions to data acquisition. AK-R made substantial contributions to revising the work. All authors contributed to the article and approved the submitted version.

## Conflict of Interest

The authors declare that the research was conducted in the absence of any commercial or financial relationships that could be construed as a potential conflict of interest.

## Abbreviations

24(S)-OHC: 24(S)-hydroxycholesterol
27-OHC: 27(S)-hydroxycholesterol
7α-OHC: 7α-hydroxycholesterol
7β-OHC: 7β-hydroxycholesterol
AD: Alzheimer’s disease
ADL: Activities of Daily Living
APP: amyloid-precursor protein
BBB: blood-brain barrier
CSF: cerebrospinal fluid
CYP46A1: 24-hydroxylase
GDS-15: 15 item version of the Geriatric Depression Scale
HDL: high-density lipoprotein
IADL: Instrumental Activities of Daily Living
LDL: low-density lipoprotein
MCI: mild cognitive impairment
MMSE: Mini-Mental State Examination
TC: total cholesterol
VaD: vascular dementia.
